# The LifeCycle Project-EU Child Cohort Network: a federated analysis infrastructure and harmonized data of more than 250,000 children and parents

**DOI:** 10.1007/s10654-020-00662-z

**Published:** 2020-07-23

**Authors:** Vincent W. V. Jaddoe, Janine F. Felix, Anne-Marie Nybo Andersen, Marie-Aline Charles, Leda Chatzi, Eva Corpeleijn, Nina Donner, Ahmed Elhakeem, Johan G. Eriksson, Rachel Foong, Veit Grote, Sido Haakma, Mark Hanson, Jennifer R. Harris, Barbara Heude, Rae-Chi Huang, Hazel Inskip, Marjo-Riitta Järvelin, Berthold Koletzko, Deborah A. Lawlor, Maarten Lindeboom, Rosemary R. C. McEachan, Tuija M. Mikkola, Johanna L. T. Nader, Angela Pinot de Moira, Costanza Pizzi, Lorenzo Richiardi, Sylvain Sebert, Ameli Schwalber, Jordi Sunyer, Morris A. Swertz, Marina Vafeiadi, Martine Vrijheid, John Wright, Liesbeth Duijts, LifeCycle Project Group, Vincent W. V. Jaddoe, Janine F. Felix, Liesbeth Duijts, Hanan El Marroun, Romy Gaillard, Susana Santos, Madelon L. Geurtsen, Marjolein N. Kooijman, Sara M. Mensink-Bout, Florianne O. L. Vehmeijer, Ellis Voerman, Martine Vrijheid, Jordi Sunyer, Mark Nieuwenhuijsen, Xavier Basagaña, Mariona Bustamante, Maribel Casas, Montserrat de Castro, Lourdes E. Cirugeda, Sílvia Fernández-Barrés, Serena Fossati, Raquel Garcia, Jordi Júlvez, Aitana C. Lertxundi, Nerea Lertxundi, Sabrina Llop, Mònica López-Vicente, Maria-Jose B. Lopez-Espinosa, Lea Maitre, Mario Murcia, Jose Lea, H. Urquiza, Charline Warembourg, Lorenzo Richiardi, Costanza Pizzi, Daniela Zugna, Maja Popovic, Elena Isaevska, Milena Maule, Chiara Moccia, Giovenale Moirano, Davide Rasella, Mark A. Hanson, Hazel M. Inskip, Chandni Maria Jacob, Theodosia Salika, Deborah A. Lawlor, Ahmed Elhakeem, Tim Cadman, Anne-Marie Nybo Andersen, Angela Pinot de Moira, Katrine M. Strandberg-Larsen, Marie Pedersen, Johan L. Vinther, John Wright, Rosemary R. C. McEachan, Paul Wilson, Dan Mason, Tiffany C. Yang, Morris A. Swertz, Eva Corpeleijn, Sido Haakma, Marloes Cardol, Esther van Enckevoort, Eleanor Hyde, Salome Scholtens, Harold Snieder, Chris H. L. Thio, Marina Vafeiadi, Lida Chatzi, Katerina C. A. Margetaki, Theano Roumeliotaki, Jennifer R. Harris, Johanna L. Nader, Gun Peggy Knudsen, Per Magnus, Marie-Aline Charles, Barbara Heude, Lidia Panico, Mathieu Ichou, Blandine de Lauzon-Guillain, Patricia Dargent-Molina, Maxime Cornet, Sandra M. Florian, Faryal Harrar, Johanna Lepeule, Sandrine Lioret, Maria Melchior, Sabine Plancoulaine, Marjo-Riitta Järvelin, Sylvain Sebert, Minna Männikkö, Priyanka Parmar, Nina Rautio, Justiina Ronkainen, Mimmi Tolvanen, Johan G. Eriksson, Tuija M. Mikkola, Berthold Koletzko, Veit Grote, Nicole Aumüller, Ricardo Closa-Monasterolo, Joaquin Escribano, Natalia Ferré, Dariusz Gruszfeld, Kathrin Gürlich, Jean-Paul Langhendries, Veronica Luque, Enrica Riva, Phillipp Schwarzfischer, Martina Totzauer, Elvira Verduci, Annick Xhonneux, Marta Zaragoza-Jordana, Maarten Lindeboom, Amelie Schwalber, Nina Donner, Rae-Chi Huang, Rachel E. Foong, Graham L. Hall, Ashleigh Lin, Jennie Carson, Phillip Melton, Sebastian Rauschert

**Affiliations:** 1 Department of Pediatrics, Erasmus MC, University Medical Center Rotterdam, The Generation R Study Group, (Na 29-18), PO Box 2040, 3000 CA Rotterdam, The Netherlands; 2 Generation R Study Group, Erasmus MC, University Medical Center Rotterdam, Rotterdam, The Netherlands; 3 Section of Epidemiology, Department of Public Health, University of Copenhagen, Copenhagen, Denmark; 4 Université de Paris, Centre for Research in Epidemiology and Statistics (CRESS), INSERM, INRAE, Paris, France; 5 ELFE Joint Unit, French Institute for Demographic Studies (Ined), French Institute for Medical Research and Health (INSERM), French Blood Agency, Aubervilliers, France; 6 Department of Preventive Medicine, Keck School of Medicine, University of Southern California, Los Angeles, CA USA; 7 Department of Epidemiology, University of Groningen, University Medical Center Groningen, Groningen, The Netherlands; 8 Concentris Research Management GmbH, Fürstenfeldbruck, Germany; 9 MRC Integrative Epidemiology Unit, University of Bristol, Bristol, UK; 10 Population Health Sciences, Bristol Medical School, University of Bristol, Bristol, UK; 11 Department of General Practice and Primary Health Care, University of Helsinki and Helsinki University Hospital, Helsinki, Finland; 12 Folkhälsan Research Center, Helsinki, Finland; 13 Obstetrics and Gynecology, Yong Loo Lin School of Medicine, National University of Singapore and National University Health System, Singapore, Singapore; 14 Singapore Institute for Clinical Sciences (SICS), Agency for Science and Technology (A*STAR), Singapore, Singapore; 15 Telethon Kids Institute, Perth, WA Australia; 16 School of Physiotherapy and Exercise Science, Curtin University, Perth, WA Australia; 17 Department of Pediatrics, Dr. von Hauner Children’s Hospital, University Hospital, LMU, Munich, Germany; 18 University of Groningen, University Medical Center Groningen, Genomics Coordination Center, Groningen, The Netherlands; 19 Institute of Developmental Sciences, Faculty of Medicine, University of Southampton, Southampton, UK; 20 NIHR Southampton Biomedical Research Centre, University of Southampton and University Hospital Southampton NHS Foundation Trust, Southampton, UK; 21 Centre for Fertility and Health, The Norwegian Institute of Public Health, Oslo, Norway; 22 Division of Health Data and Digitalization, Norwegian Institute of Public Health, Oslo, Norway; 23 MRC Lifecourse Epidemiology Unit, University of Southampton, Southampton General Hospital, Southampton, UK; 24 Center for Life-Course Health Research, Faculty of Medicine, University of Oulu, Oulu, Finland; 25 Department of Epidemiology and Biostatistics, MRC-PHE Centre for Environment and Health, School of Public Health, Imperial College London, London, UK; 26 Department of Life Sciences, College of Health and Life Sciences, Brunel University London, London, UK; 27 Unit of Primary Health Care, Oulu University Hospital, OYS, Oulu, Finland; 28 NIHR Bristol Biomedical Research Centre, Bristol, UK; 29 Department of Economics, VU University Amsterdam, Amsterdam, The Netherlands; 30 Bradford Institute for Health Research, Bradford Teaching Hospitals NHS Foundation Trust, Bradford, UK; 31 Department of Genetics and Bioinformatics, Division of Health Data and Digitalisation, Norwegian Institute of Public Health, Oslo, Norway; 32 Cancer Epidemiology Unit, Department of Medical Sciences, University of Turin, Turin, Italy; 33 ISGlobal, Barcelona, Spain; 34 Universitat Pompeu Fabra (UPF), Barcelona, Spain; 35 CIBER Epidemiología y Salud Pública (CIBERESP), Barcelona, Spain; 36 IMIM (Hospital del Mar Medical Research Institute), Barcelona, Spain; 37 Department of Genetics, University of Groningen, University Medical Center Groningen, Groningen, The Netherlands; 38 Department of Social Medicine, Faculty of Medicine, University of Crete, Heraklion, Crete, Greece

**Keywords:** Consortium, Birth cohorts, Exposome, Life course, Non-communicable diseases

## Abstract

Early life is an important window of opportunity to improve health across the full lifecycle. An accumulating body of evidence suggests that exposure to adverse stressors during early life leads to developmental adaptations, which subsequently affect disease risk in later life. Also, geographical, socio-economic, and ethnic differences are related to health inequalities from early life onwards. To address these important public health challenges, many European pregnancy and childhood cohorts have been established over the last 30 years. The enormous wealth of data of these cohorts has led to important new biological insights and important impact for health from early life onwards. The impact of these cohorts and their data could be further increased by combining data from different cohorts. Combining data will lead to the possibility of identifying smaller effect estimates, and the opportunity to better identify risk groups and risk factors leading to disease across the lifecycle across countries. Also, it enables research on better causal understanding and modelling of life course health trajectories. The EU Child Cohort Network, established by the Horizon2020-funded LifeCycle Project, brings together nineteen pregnancy and childhood cohorts, together including more than 250,000 children and their parents. A large set of variables has been harmonised and standardized across these cohorts. The harmonized data are kept within each institution and can be accessed by external researchers through a shared federated data analysis platform using the R-based platform DataSHIELD, which takes relevant national and international data regulations into account. The EU Child Cohort Network has an open character. All protocols for data harmonization and setting up the data analysis platform are available online. The EU Child Cohort Network creates great opportunities for researchers to use data from different cohorts, during and beyond the LifeCycle Project duration. It also provides a novel model for collaborative research in large research infrastructures with individual-level data. The LifeCycle Project will translate results from research using the EU Child Cohort Network into recommendations for targeted prevention strategies to improve health trajectories for current and future generations by optimizing their earliest phases of life.

**Electronic supplementary material:**

The online version of this article (10.1007/s10654-020-00662-z) contains supplementary material, which is available to authorized users.

## Rationale

Early life seems to be an important window of opportunity to improve health across the full lifecycle. An accumulating body of evidence suggests that exposure to adverse stressors during early life leads to developmental adaptations, which subsequently affect disease risk in later life [1]. Moreover, geographical, socio-economic, and ethnic differences are related to health inequalities from early life onwards [1]. These research findings suggest that optimizing early-life conditions has the yet unfulfilled potential to improve life course health trajectories for individuals themselves and also for their offspring through transgenerational effects [2]. A better understanding of the causality, pathways and life course health trajectories explaining associations of early-life stressors with later life disease is urgently needed to translate results from observational studies into population-health prevention strategies.

Many European pregnancy and childhood cohorts have been established over the last years to assess the associations of early life with health across the lifecycle [3]. These cohorts are invaluable resources to obtain insight into societal, environmental, lifestyle and nutrition related determinants that may influence the onset and evolution of risk factors and diseases in later life. Cohort studies that started during pregnancy or early childhood provide the unique opportunity to study the potential for early-life interventions on factors that cannot be easily studied in experimental settings, such as socio-economic, migration, urban environment and lifestyle related determinants. Data from cohort studies can also be used for advanced analytical approaches such as sibling analyses and Mendelian randomization to assess causality of observed associations [4].

The impact of these cohorts and their data could be strongly increased by combining data from different cohorts. Combining data will lead to larger numbers and the opportunity to better identify risk groups and risk factors leading to disease across the lifecycle [3]. Also, it enables research for a better causal understanding and modelling of life course health trajectories. The enormous wealth of high-quality prospective cohort studies enables collaboration at individual participant data level. Meta-analyzing individual participant data has the advantage that it can identify smaller effect estimates, specific subgroups, and mediator effects and, maybe most importantly, capitalizes on existing published and unpublished data. Results from well-performed individual participant data meta-analyses suffer less from publication bias than meta-analyses based on published data. Multiple individual participant data meta-analyses on environmental exposures, lifestyle related and (epi)genetic associations have already been published as part of birth cohort collaborations [5–22].

**Fig. 1:**
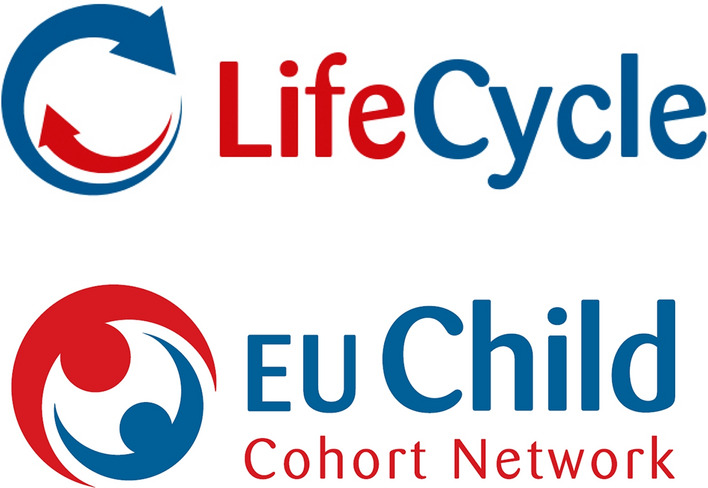
Logo’s of the LifeCycle Project and EU Child Cohort Network

The LifeCycle Project is a Horizon 2020-funded (2017–2022) international project. The general objective of the LifeCycle Project is to bring together pregnancy and childhood cohort studies into a new, open and sustainable EU Child Cohort Network, to use this network for identification of novel markers of early-life stressors affecting health trajectories throughout the life course, and to translate findings into policy recommendations for targeted prevention strategies. The overall concepts, design and future perspectives are described in this paper. The logos of the LifeCycle Project are given in Fig. 1.

## The EU Child Cohort Network

**Fig. 2:**
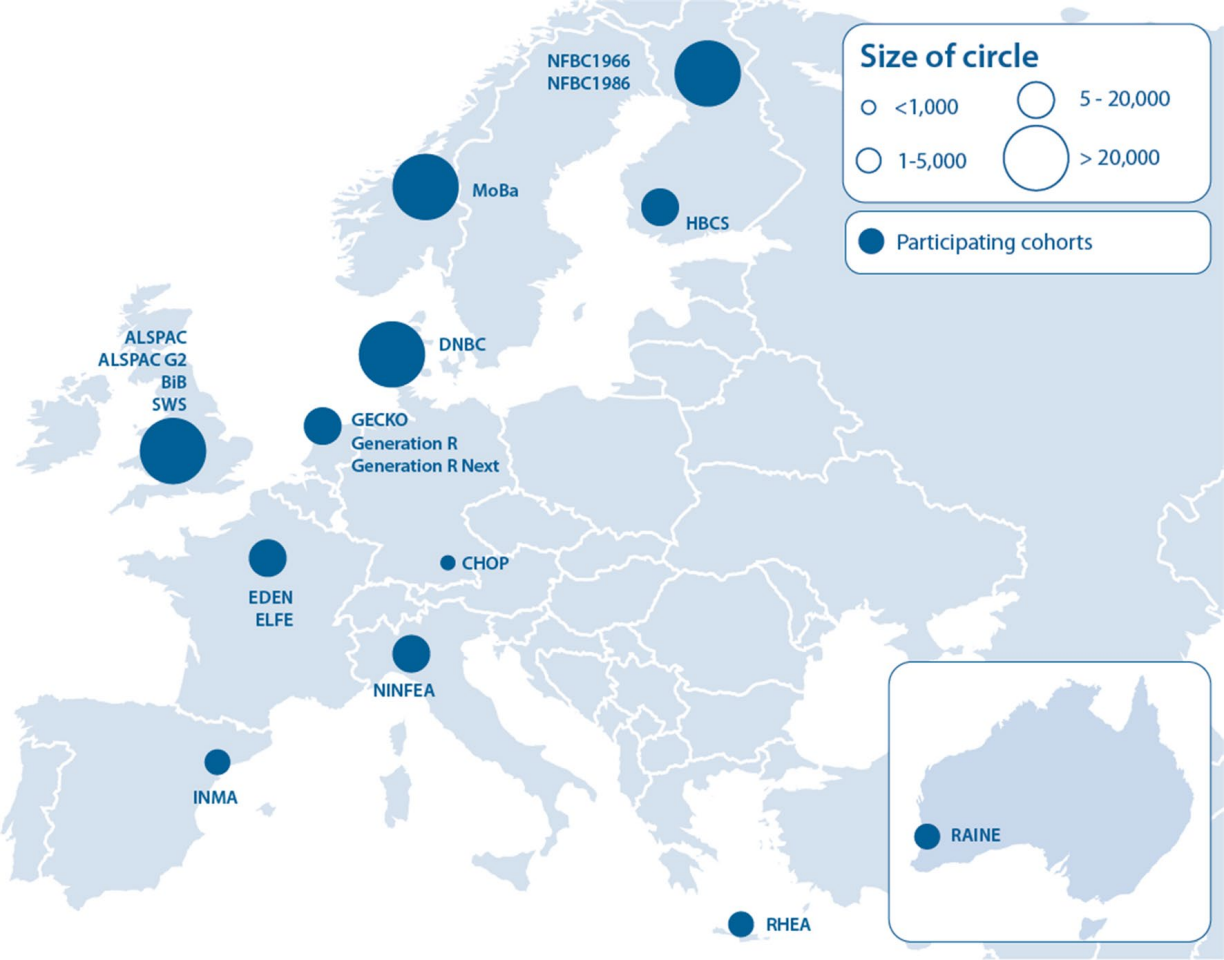
LifeCycle Project core cohorts that established the EU Child Cohort Network

The EU Child Cohort Network, the main deliverable of the LifeCycle Project, brings together nineteen pregnancy and childhood cohorts. Together, they include more than 250,000 children and their parents (Fig. 2; Table 1). Recruitment to the cohorts of the EU Child Cohort Network began prior to and during pregnancy, as well as in childhood; together, the follow-up of these cohorts span the full life course and contain detailed phenotypic information and biological samples. The research potential of the EU Child Cohort Network is summarized in Table 2. The EU Child Cohort Network should be operational mid-2020. This network is open for other partners with population-based cohorts that started in early life and will be sustainable after the duration of the Horizon 2020 funded LifeCycle Project. The EU Child Cohort Network could contribute to future collaborations between different cohorts.

**Table 1:** LifeCycle Project cohorts that together form the basis of the EU Child Cohort Network

Cohort, Country (N)	Design, birth years, Follow-up Main early-life stressors	Available mediators	Available outcomes
ALSPAC United Kingdom N = 14,500 [74, 75]	Prospective, 1991–1992 Pregnancy-25 yrs Socio-economic, migration, and life-style determinants, genome wide association screen	Epigenetics Metabolomics Allergy Brain development by MRI	Cardio-metabolic: BMI, blood pressure, cardiac structure and function, lipids, insulin, glucose Respiratory: wheezing, infections, asthma, lung function Mental: behaviour, cognition, education, ASD, ADHD, anxiety, depression
ALSPAC-G2 United Kingdom N = 2000 [76]	Prospective, from 2011 Preconception-2 yrs Socio-economic, migration and life-style determinants	Epigenetics Metabolomics Brain development by ultrasound	Cardio-metabolic: BMI, blood pressure Respiratory: wheezing, asthma Mental: behaviour, cognition
BIB United Kingdom N = 11,000 [77]	Prospective, 2007–2011 Pregnancy-9 yrs Socio-economic, migration, urban environment, and life-style determinants, genome wide association screen	Epigenetics Metabolomics Allergy Brain development by ultrasound	Cardio-metabolic: BMI, blood pressure, lipids, insulin, glucose Respiratory: wheezing, infections, asthma, lung function Mental: behaviour, cognition, education, ASD, ADHD, anxiety, depression
CHOP Germany N = 500 [78]	Prospective, 2002–2004 Pregnancy-11 yrs Socio-economic, life-style determinants, genome wide association screen	Epigenetics Metabolomics, Allergy	Cardio-metabolic: BMI, blood pressure, cardiac structure and function, lipids, insulin, glucose Respiratory: wheezing, asthma Mental: behaviour, cognition
DNBC Denmark N = 70,000 [79]	Prospective, 1996–2002 Pre-pregnancy-20 yrs Socio-economic, migration, urban environment, and life-style determinants, genome wide association screen	Allergy	Cardio-metabolic: BMI Respiratory: wheezing, infections, asthma, lung function Mental: behaviour, cognition, education, ASD, ADHD, anxiety, depression
EDEN France N = 2000 [80]	Prospective, 2003–2005 Pre-school-15 yrs Socio-economic, migration and life-style determinants	Allergy	Cardio-metabolic: BMI, blood pressure, lipids, insulin, glucose Respiratory: wheezing, lung function, asthma. Mental: behaviour, cognition, education
ELFE France N = 18,000 [81]	Prospective, 2011 Pre-school-5 yrs Socio-economic, migration, urban environment, life-style determinants	Allergy	Cardio-metabolic: BMI Respiratory: wheezing, infections, asthma Mental: behaviour, cognition
GECKO the Netherlands N = 2500 [82]	Prospective, 2006–2007 Pregnancy-10 yrs Socio-economic, migration, life-style	Allergy	Cardio-metabolic: BMI, blood pressure Respiratory: wheezing, asthma Mental: behaviour, education
Generation R the Netherlands N = 7000 [83, 84]	Prospective, 2002–2006 Pregnancy-17 yrs Socio-economic, migration, urban environment, and life-style determinants, genome wide association screen	Epigenetics Metabolomics Allergy Brain development by ultrasound/MRI	Cardio-metabolic: BMI, blood pressure, cardiac structure and function, lipids, insulin, glucose Respiratory: wheezing, infections, lung function, asthma Mental: behaviour, cognition, education, ASD, ADHD, anxiety, depression
Generation R Next the Netherlands N = 2000	Prospective, 2016–2019 Pre-pregnancy-2 yrs Socio-economic, migration, urban environment, and life-style determinants	Epigenetics Metabolomics Allergy Brain development by ultrasound/MRI	Cardio-metabolic: body mass index, blood pressure, cardiac structure and function, lipids, insulin, glucose Respiratory: wheezing, infections, lung function, asthma Mental: behaviour, cognition
HBCS Finland N = 13,000 [85]	Longitudinal, 1934–1944 Pregnancy-80 yrs Socio-economic, migration, and life-style determinants, genome wide association screen		Cardio-metabolic: BMI, blood pressure, lipids, insulin, glucose, hypertension, type 2 diabetes, dyslipidaemia Respiratory: asthma, COPD Mental: cognition, psychiatric illness
INMA Spain N = 3500 [86]	Prospective, 1997–2008 Pregnancy-20 yrs Socio-economic, migration, urban environment, and life-style determinants, genome wide association screen	Epigenetics Metabolomics Allergy	Cardio-metabolic: BMI, blood pressure, lipids, insulin, glucose Respiratory: wheezing, respiratory infections, lung function, asthma Mental: behaviour, cognition, ASD, ADHD, anxiety, depression
MoBa Norway N = 90,000 [87]	Prospective, 1999–2008 Pregnancy-14 yrs Socio-economic, urban environment and life-style determinants, genome wide association screen	Epigenetics Allergy Brain development by MRI	Cardio-metabolic: BMI, blood pressure Respiratory: wheezing, respiratory infections, asthma Mental: behaviour, cognition, ASD, ADHD, anxiety, depression
NFBC1966 Finland N = 12,000 [88]	Prospective, 1966 Pregnancy-50 yrs Socio-economic, migration, life-style determinants, genome wide association screen	Epigenetics Metabolomics Allergy Brain development by MRI	Cardio-metabolic: BMI, blood pressure, cardiac structure and function, lipids, insulin, glucose Respiratory: wheezing, respiratory infections, lung function, asthma, COPD Mental: behaviour, cognition, education, ASD, ADHD, anxiety, depression
NFBC1986 Finland N = 9500 [89]	Prospective, 1986 Pregnancy-30 yrs Socio-economic, migration, urban environment, and life-style determinants, genome wide association screen	Epigenetics Metabolomics Allergy Brain development by MRI	Cardio-metabolic: BMI, blood pressure, cardiac structure and function, lipids, insulin, glucose Respiratory: wheezing, respiratory infections, lung function, asthma, COPD Mental: behaviour, cognition, education, ASD, ADHD, anxiety, depression
NINFEA Italy N = 7500 [90]	Prospective, 2005–2016 Pregnancy-13 yrs Socio-economic, urban environment, and life-style determinants	Allergy	Cardio-metabolic: BMI Respiratory: wheezing, respiratory infections, asthma Mental: behaviour, education
RAINE Australia N = 2900 [91]	Prospective, 1989–1992 Pregnancy-25 yrs Socio-economic, migration, urban environment, and life-style determinants, genome wide association screen	Epigenetics Metabolomics Allergy Brain development	Cardio-metabolic: BMI, blood pressure, lipids, insulin, glucose Respiratory: wheezing, respiratory infections, lung function, asthma Mental: behaviour, cognition, education, ASD, ADHD, anxiety, depression
RHEA Greece N = 1300 [92]	Prospective, 2007–2008 Pregnancy-7 yrs Socio-economic, migration, urban environment, and life-style determinants	Epigenetics Metabolomics Allergy	Cardio-metabolic: BMI, blood pressure, lipids, insulin, glucose Respiratory: wheezing, respiratory infections, lung function, asthma Mental: behaviour, cognition, education, ASD, ADHD, anxiety, depression
SWS United Kingdom N = 3000 [93]	Prospective, 1998–2007 Prepregnancy-18 yrs Socio-economic and life-style determinants	Allergy	Cardio-metabolic: BMI, blood pressure, cardiac function and structure Respiratory: wheezing, respiratory infections, lung function, asthma Mental: behaviour, cognition, education, anxiety, depression

**Table 2:** Potential of the LifeCycle Project-EU Child Cohort Network

*Collaboration between prospective pregnancy/child cohort studies offers the opportunities to*	1
Perform analyses in over 250,000 children and their parents	
Harmonize methods for data collection, biobanks, management, and analyses	
Perform analyses on published and unpublished data which limits publication bias	
Perform individual participant data meta-analyses with better statistical precision	
Stratify groups by geographical area or sex	
Compare determinants and outcomes between European populations	
Examine consequences of small variations in determinants from early life onwards	
Identify variations in geography and time periods for specific associations	
Infer causality from observed associations by advanced analytical approaches	
Enable analyses on life course trajectories on risk factors of non-communicable diseases	
Explore different life course models	

The LifeCycle Project and its EU Child Cohort Network do not stand on their own. By building on and collaborating with existing initiatives, we will create new synergies and form the basis of future initiatives. These synergies bring together principal investigators and their expertise of several international collaborations. These initiatives include:

- Cohort collaboration and data sharing platforms: BioSHaRe [23], CHICOS [24], DataSHIELD [25], DynaHEALTH [26], EarlyNutrition [27], ENRIECO [28], HELIX [29, 30], InterConnect [31] and NutriMenthe [32] (all EU-FP6, FP7 projects or Horizon2020).
- Genetic and epigenetic collaborations: Early Growth & Longitudinal Epidemiology (EAGLE), Early Growth Genetics (EGG) [33], Pregnancy And Childhood Epigenetics (PACE) [34] (no specific funds for the collaboration).
- E-Learning: Early Nutrition Academy [35] (EU-FP7 project).

## Data harmonisation

**Fig. 3:**
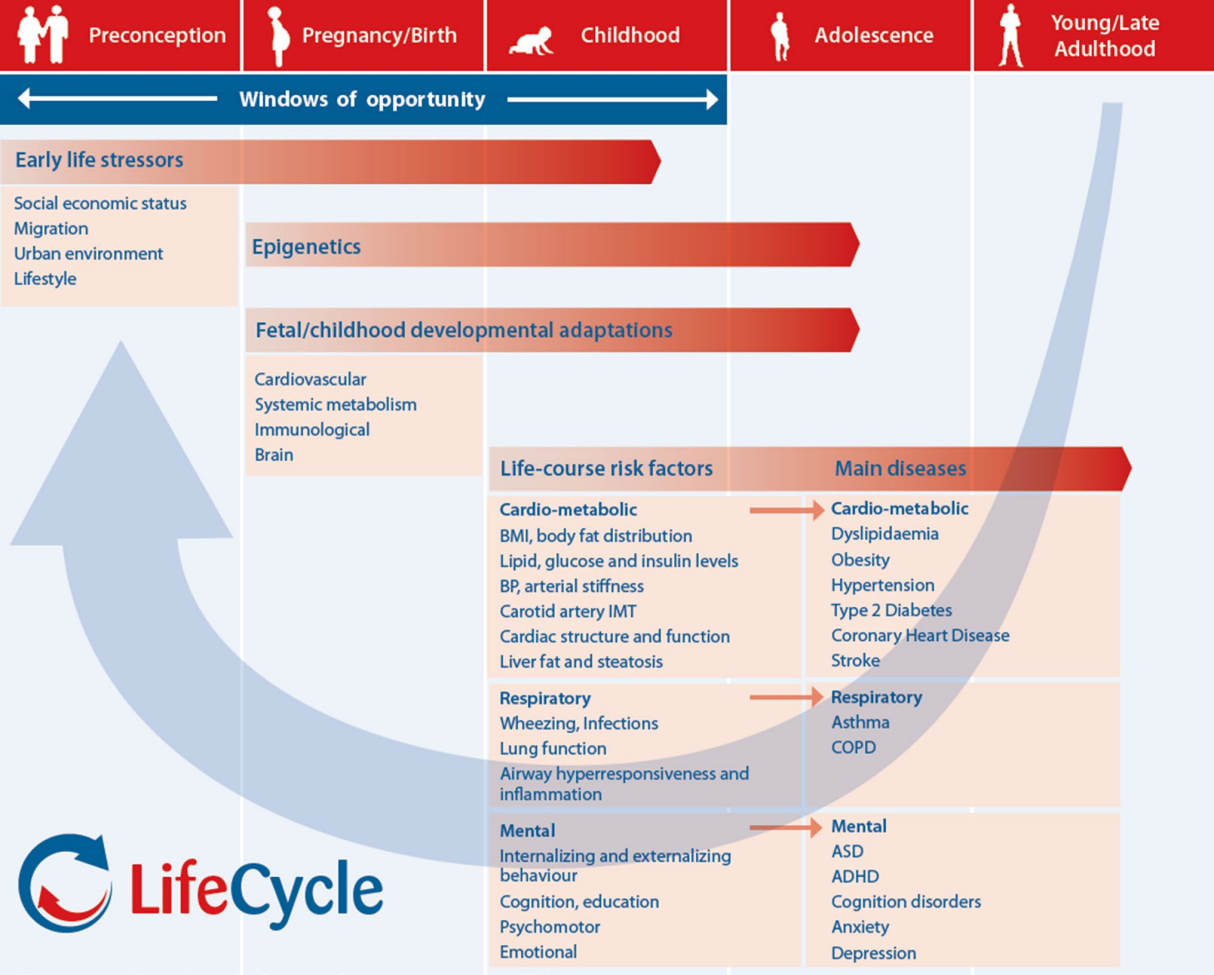
Main concepts of the LifeCycle Project and related data in the EU Child Cohort Network

The LifeCycle Project has developed a harmonized set of variables in each cohort necessary to perform multi-cohort analyses on different research questions. The harmonization work is performed by a data-harmonization group with representatives from each partner or cohort. Based on the primary research focus in the LifeCycle Project, a priority list of variables has been developed for harmonisation. The cohort studies participating in the EU Child Cohort Network will be further enriched with novel harmonized integrated data on early-life stressors related to socio-economic, migration, urban environment and lifestyle determinants, based on data availability within the cohorts and external data from registries [36]. Integrated data will also be used to construct a novel holistic ‘dynamic early-life exposome’ model, which will encompass many human environmental exposures during various stages of early life [37–40]. The harmonized variables relate to the main research hypotheses (Fig. 3), and include:

- Main exposures: Socioeconomic, migration, urban environment, lifestyle and nutrition related factors, genome-wide association screen;
- Main mediators: Epigenetics, metabolomics, allergy, brain development;
- Main outcomes: Cardio-metabolic (body mass index (BMI), body composition, blood pressure, cardiac structure and function, lipids, insulin, glucose); respiratory (allergy, wheezing, infections, lung function, asthma), mental (behaviour, cognition, education, ASD, ADHD, anxiety, depression);

The availability of these data in different cohorts is given in Table 1.

## Federated data analysis approach

**Fig. 4:**
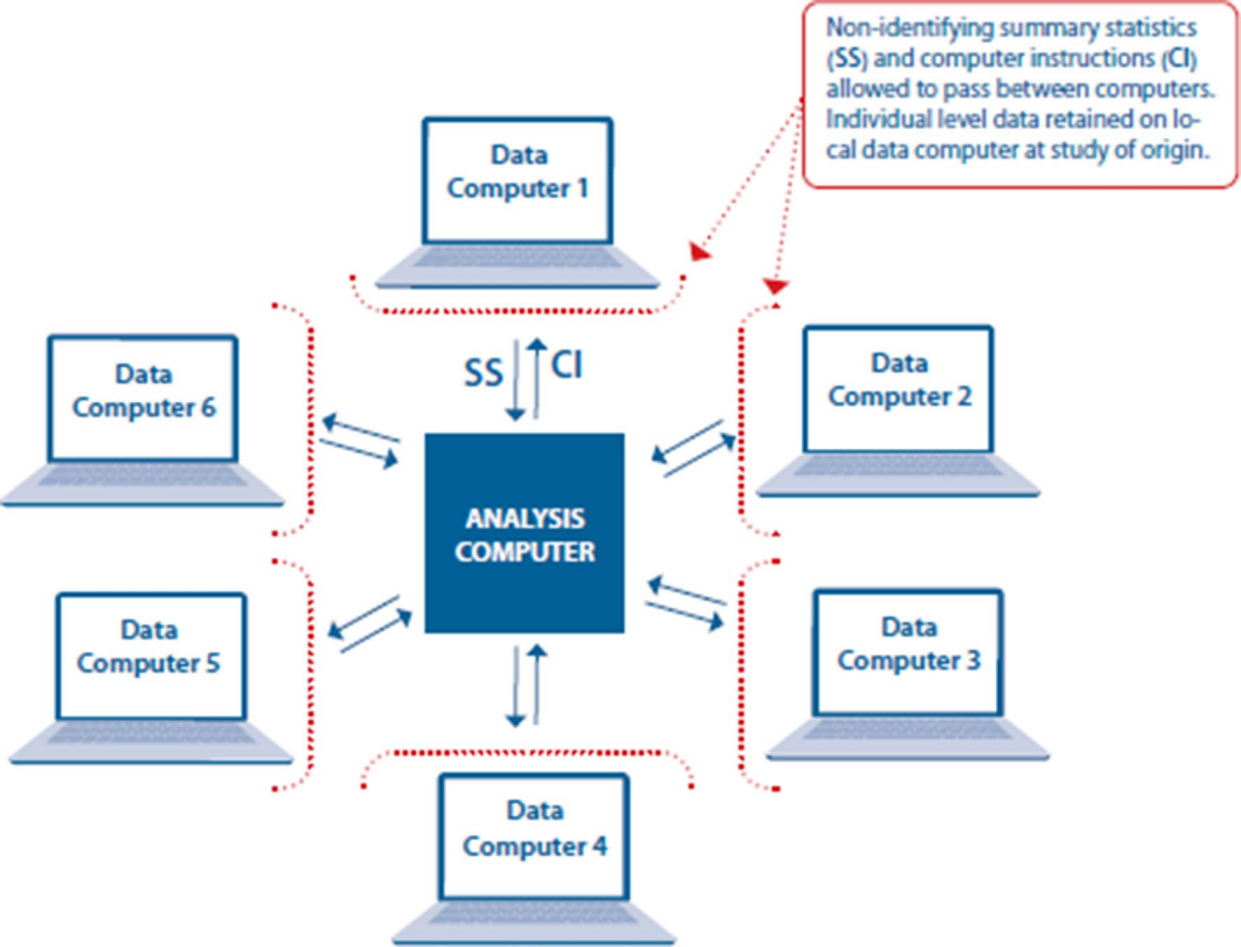
Federated analysis approach using DataSHIELD approach

Analyses in the EU Child Cohort will be predominantly using DataSHIELD, developed as part of the EU-FP7 BioSHaRe Project [23, 25]. This is a safe and robust data analysis platform to perform joint multisite individual participant data meta-analyses, without physically transferring data (Fig. 4). DataSHIELD enables connections between local servers to analyze harmonized data located at different institutes. The major advantage of this approach is that the data from the different institutes, which together form the EU Child Cohort Network, are accessible for different researchers from various sites whilst they remain at the local sites.

## Fair principles

The EU Child Cohort Network data management and access are based on the following key principles:

- Full compliance with best practice in data privacy and security;
- Use of coded data with appropriate institutional and participant consent;
- Use of privacy enhancing technologies such as filters;
- Use of policies that enable greater use of data in research;
- Approval of all procedures, policies and methods by the relevant local authorities.

Management of and access to all data is primarily the responsibility of each institution. The FAIR (findable, accessible, interoperable, reusable) principles are taken into account for the general data management approach.

### Findable

The LifeCycle Project has revitalized the existing www.birthcohorts.net website. This website gives an overview of pregnancy and birth cohorts and the data available in these cohorts. Specific details of variables included in the EU child cohort network and their availability in the cohorts are presented in the open access EU Child Cohort Network Variable Catalogue. The catalogue was built using the MOLGENIS software platform for scientific data extending on BBMRI-ERIC directory of biobanks [41, 42]. It also documents how each cohort has harmonized these variables, including information about the source variables used by the cohorts. No actual data are given in the online catalogue. All relevant websites and their contents are presented in Table 3.

**Table 3:** Websites of the LifeCycle Project–EU child cohort network

Data related to the LifeCycle Project is findable through different websites
**LifeCyce Project**
https://lifecycle-project.eu website
Overview of the LifeCycle Project
All protocols for harmonisation and setting up the data-servers
Open access
Links to other relevant websites
**Birthcohorts.net**
www.birthcohorts.net
Overview of all cohorts and their data
Open access, no restriction for access on cohort information
**EU Child Cohort Network Variable Catalogue**
http://catalogue.lifecycle-project.eu
Overview of harmonized data and variables in each cohort
Open access
Find function is included in website
**EU Child Cohort harmonized data**
Cohort websites via www.lifecycle-project.eu
Harmonized data from different cohorts
Data server is within institutional firewall
Access to data can only be given by data owner (LifeCycle Project partner)

### Accessible

A harmonized set of data for EU Child Cohort Network is available by a server controlled by or located at each specific institute. Harmonized data from each cohort are held on secure Opal servers (http://opaldoc.obiba.org/en/latest/) at their institution. Protocols for setting up this data infrastructure are available, together with YouTube instruction videos. Data are accessed via a central analysis server using the R-based platform DataSHIELD. Access to data is conditional on approval by the cohort. Partners and their cohorts can always decide to share research data without using DataSHIELD, conditional on relevant local ethical and legal approvals. This approach is used for analyses that are not yet possible in DataSHIELD [25]. The field of data sharing and cross study analyses is rapidly advancing. Although we start with using DataSHIELD, we recognise that over time this may change.

### Interoperable

Existing data have been harmonized and integrated into exposure variables to make them interoperable. Protocols for harmonization are available online. All harmonized data from different cohorts have been renamed into standardized variable names. A full list of the available variables per cohort is available in the EU Child Cohort Network Variable Catalogue.

### Reusable

The EU Child Cohort Network reuses data that are already available within cohorts. The EU Child Cohort Network, with the harmonized set of variables and infrastructure, should be sustainable beyond the duration of the LifeCycle Project. During the last two years, four other European consortia have been funded, which are planning to build upon the harmonized data and federated analysis infrastructure in the EU Child Cohort Network. These consortia include the EUCAN-Connect, NutriPROGRAM, ATHLETE and LongITools Projects. Future collaborations may include not only European, but also global initiatives such as the NIH-Environmental influences on Child Health Outcomes (ECHO) Programme in the United States, which aims to build a virtual paediatric cohort based on new and existing birth cohorts, recognizing the enormous opportunities in optimizing and networking existing resources [43, 44].

## Data governance

The LifeCycle Project or EU Child Cohort Network do not own data, but bring data from other cohorts together via a federated data analysis platform. Ethical and legal responsibility for data management and security is maintained by the source studies or home institutions. The principal investigators or home institutions should always administer permission for external access to specific data on their server for addressing research questions. The EU Child Cohort Network cannot provide open access to researchers. The data sharing protocols and agreements will be updated regularly, according to new legal practices, such as the European General Data Protection Regulation 2016/679 (GDPR). All governance protocols will take not only the short-term, but also the long-term EU Child Cohort Network, beyond the LifeCycle Project duration, into account.

##  EU Child Cohort Network research proposals

Proposals for research using the EU Child Cohort Network can be put forward by both LifeCycle Project partners and other researchers. External researchers can send a request for EU Child Cohort Network data use to the participating cohorts or lifecycle@erasmusmc.nl. Each LifeCycle Project proposal is discussed in the relevant coordinating work package (https://lifecycle-project.eu/for-scientists/workpackages/) and subsequently distributed among all cohorts participating in the LifeCycle Project and EU Child Cohort Network. Cohorts can opt-in or opt-out of each analysis, depending on the data availability, research interests or involvement in other projects. In the first phase, the focus of research projects is on those projects related to the LifeCycle Project research aims (see below). An efficient governance structure was organized and agreed upon by researchers and ethical and legal representatives. EU Child Cohort Network governance structure will be updated regularly where needed and will be made sustainable after the LifeCycle Project duration. Because there is no physical transfer of data needed, we are currently exploring the possibility of working with a short Data Access Agreement that replaces commonly used Data Transfer Agreements. When the EU Child Cohort Network is fully operational we aim to have regular EU Child Cohort Network meetings or telephone conferences to discuss:

- Research projects (novel proposals, progress of ongoing projects);
- Harmonization (novel proposals, progress of ongoing efforts);
- DataSHIELD analysis approaches (priorities for further development);
- Any relevant ethical or legal issues concerning federated analysis approaches;

Participants in these meetings or telephone conferences are not only LifeCycle Project Partners, but representatives of all institutes that have harmonized their data and set up the IT infrastructure needed for the federated analysis of data via DataShield.

## LifeCycle Project primary research areas

The LifeCycle Project uses the integrated and harmonized set of variables from the EU Child Cohort Network for identification of early-life stressors influencing cardio-metabolic, respiratory and mental developmental adaptations and health trajectories during the full life course (Fig. 3).

### Integrated early-life stressors approach and the exposome

Early-life stressors, including socio-economic, migration, urban environmental, and lifestyle related factors, have been associated with cardio-metabolic, respiratory, and mental health and disease, which together contribute greatly to the global burden of non-communicable diseases [5–22]. An accumulating body of evidence suggests that exposure to these factors during fetal life and childhood affects later life health trajectories [38]. Thus far, studies focused on the effects of early-life environmental exposures on later life health outcomes have largely been using a ‘one-exposure at one-time point’ approach. Research from LifeCycle Project partners suggests that instead of exposure to single stressors that individually may have weak effects, exposure to a cluster or pattern of adverse early-life stressors in specific age windows is more likely to influence health during the lifecycle [39]. We will apply a holistic ‘early-life exposome’ model to encompass many human environmental exposures, which is dynamic from conception onwards and complements the genome. To develop this early-life exposome, we will specifically take into account measurements in the external environment (socio-economic, migration, urban environment, and lifestyle factors), and biological markers reflecting the internal environment (DNA methylation, RNA expression, and metabolomics), and the dynamic life course nature of the exposome. We will use available methods developed as part of the EU-FP7 HELIX Project for further development of the early-life exposome model [29].

### Cardio-metabolic, respiratory and mental health outcomes

Embryonic life, fetal life and early childhood are characterized by high developmental rates and seem to be critical periods for developmental adaptations with long-term consequences. Research from LifeCycle Project partners have shown that specific maternal lifestyle factors and fetal growth variation in early pregnancy are related to non-communicable diseases and their risk factors [45–49]. We will use repeatedly measured exposure, mediator and outcome data from the EU Child Cohort Network to compare different potential life course models including those assuming specific critical periods and those assuming interactive and cumulative effects throughout the life course. We will relate early-life stressors measured in different early-life periods (preconception, fetal life, early childhood) with life course health trajectories. We specifically hypothesize that early-life stressors lead to developmental adaptations of:

- The cardiovascular system assessed in detail by advanced cardiac and great vessel ultrasound or Magnetic Resonance Imaging (MRI), and systemic metabolism, detected by measuring hundreds of metabolites using high-throughput approaches, which precede the development of cardio-metabolic diseases [50–60].
- Lung volumes, airway patency assessed by lung function measurements and clinical assessments, and immunological or allergy-related assessments, which precede the development of respiratory disease [61–63].
- Structural and functional brain development assessed by ultrasound in fetal life or early infancy, or brain MRI in later life, which precede the development of mental health outcomes [64–67].

### Epigenetic pathways

An accumulating body of evidence suggests that epigenetic changes play a key role in the associations of early-life stressors with lifecycle health and disease trajectories [68]. DNA methylation, the most frequently studied epigenetic phenomenon in large populations, is a dynamic process, which may be influenced by environmental stressors such urban environment, dietary factors and smoking [68]. DNA methylation changes are more common in early life. LifeCycle Project partners have identified DNA methylation markers related to specific early-life stressors including maternal BMI, smoking, dietary factors and birth weight [12, 17]. The EU Child Cohort Network brings together many pregnancy and childhood cohorts with information about epigenome-wide DNA methylation. Availability of repeatedly measured DNA methylation and of RNA expression data enables studies on persistence and functionality of DNA methylation markers potentially involved in early-life programming of non-communicable diseases.

### Population impact

The concept that early life is critical for health and disease throughout the life course is well-acknowledged. However, there is still not much evidence for effective prevention or intervention strategies using early life as a window of opportunity to maximize the human developmental potential during the full life course. We will use different approaches to translate findings into population health recommendations. These include causal inference, aggregation of evidence for interventions based on reviews, dynamic microsimulation, and development of prediction models.

Causality cannot be directly concluded from observational studies. Advanced analytical approaches that can help to infer causality include sibling comparison studies, propensity score matching and Mendelian randomization studies, in which genetic variants are used as unconfounded proxies for adverse exposures [69]. The EU Child Cohort Network facilitates integration of different causal inference methods and comparison of their findings, which will strengthen causal inference needed for translation of findings from observational studies to public health recommendations.

We will review and summarize evidence based on findings both from observational studies in the EU Child Cohort Network and from published intervention studies to develop recommendations for population and subgroup-specific interventions focused on the earliest phases of life. Dynamic microsimulation modelling using data from cohort studies enables policy evaluations and scenario analyses focused on early-life interventions when experimental studies are not possible [70, 71]. The EU Child Cohort Network provides a unique infrastructure for these analyses, because of the available data and variation in exposures and outcomes, life course trajectories of non-communicable diseases and various subpopulations with different baseline risks.

Data from observational studies can help to develop  models to predict risk factors for non-communicable diseases. Previous studies suggested that pregnancy, birth and infancy characteristics have the potential to identify groups at risk for obesity [72, 73]. The EU Child Cohort Network is the ideal platform to develop models to predict from early-life stressor data the onset of risk factors for cardio-metabolic, respiratory and mental disease across the lifecycle. Models can include various background characteristics, which enable baseline risk estimation from socio-economic, migration, environment and lifestyle stressors, which may be difficult to modify in the short-term but help to predict the outcomes of interest.

Finally, we will develop E-learning modules and eHealth applications that will be made widely available to make the knowledge and research findings available for educational and health care purposes.

## Conclusion

The LifeCycle Project and its EU Child Cohort Network lead to great opportunities for researchers to combine harmonized data from different cohorts by a federated analysis platform. It also provides a novel model for collaborative research in large research infrastructures with individual level data. The LifeCycle Project will translate results from research using the EU Child Cohort Network into recommendations for targeted prevention strategies to improve health trajectories for current and future generations by optimizing their earliest phases of life.

## Electronic supplementary material

Below is the link to the electronic supplementary material.

Supplementary material 1 (DOCX 25 kb)

## The LifeCycle Project Group

Vincent W.V. Jaddoe^1,2^, Janine F. Felix^1,2^, Liesbeth Duijts^1,2^, Hanan El Marroun^1,3,4^, Romy Gaillard^1,2^, Susana Santos^1,2^, Madelon L. Geurtsen^1,2^, Marjolein N. Kooijman^1,2^, Sara M. Mensink-Bout^1,2^, Florianne O.L. Vehmeijer^1,2^, Ellis Voerman^1,2^, Martine Vrijheid^5,6,7^, Jordi Sunyer^5,6,7,8^, Mark Nieuwenhuijsen^5,6,7^, Xavier Basagaña^5,6,7^, Mariona Bustamante^5,6,7^, Maribel Casas^5,6,7^, Montserrat de Castro^5,6,7^, Lourdes Cirugeda^5,6,7^, Sílvia Fernández-Barrés^5,6,7^, Serena Fossati^5,6,7^, Raquel Garcia^5,6,7^, Jordi Júlvez^5,6,9^, Aitana Lertxundi^5,10,11^, Nerea Lertxundi^10,11^, Sabrina Llop^5,12^, Mònica López-Vicente^2,3,6^, Maria-Jose Lopez-Espinosa^5,12,13^, Lea Maitre^6^, Mario Murcia^12,14^, Jose Urquiza^5,6,7^, Charline Warembourg^5,6,7^, Lorenzo Richiardi^15^, Costanza Pizzi^15^, Daniela Zugna^15^, Maja Popovic^15^, Elena Isaevska^15^, Milena Maule^15^, Chiara Moccia^15^, Giovenale Moirano^15^, Davide Rasella^15^, Mark A Hanson^16,17^, Hazel M. Inskip^17,18^, Chandni Maria Jacob^16,17^, Theodosia Salika^18^, Deborah A. Lawlor^19,20,21^, Ahmed Elhakeem^19,21^, Tim Cadman^19,21^, Anne-Marie Nybo Andersen^22^, Angela Pinot de Moira^22^, Katrine Strandberg-Larsen^22^, Marie Pedersen^22^, Johan L Vinther^22^, John Wright^23^, Rosemary R.C. McEachan^23^, Paul Wilson^24^, Dan Mason^23^, Tiffany C. Yang^23^, Morris A. Swertz^25,26^, Eva Corpeleijn^27^, Sido Haakma^25^, Marloes Cardol^27^, Esther van Enckevoort^25,26^, Eleanor Hyde^25,26^, Salome Scholtens^25,26^, Harold Snieder^27^, Chris H.L. Thio^27^, Marina Vafeiadi^28^, Lida Chatzi^29^, Katerina Margetaki^29^, Theano Roumeliotaki^28^, Jennifer R. Harris^30,31^, Johanna L. Nader^32^, Gun Peggy Knudsen^33^, Per Magnus^30^, Marie-Aline Charles^34,35^, Barbara Heude^34^, Lidia Panico^36^, Mathieu Ichou^36^, Blandine de Lauzon-Guillain^34^, Patricia Dargent-Molina^34^, Maxime Cornet^34^, Sandra M. Florian^36^, Faryal Harrar^34^, Johanna Lepeule^37^, Sandrine Lioret^34^, Maria Melchior^38^, Sabine Plancoulaine^34^, Marjo-Riitta Järvelin^39,40,41,42^, Sylvain Sebert^39^, Minna Männikkö^43^, Priyanka Parmar^39^, Nina Rautio^39^, Justiina Ronkainen^39^, Mimmi Tolvanen^39^, Johan G Eriksson^44,45,46,47^, Tuija M. Mikkola^45^, Berthold Koletzko^48^, Veit Grote^48^, Nicole Aumüller^48^, Ricardo Closa-Monasterolo^49^, Joaquin Escribano^49^, Natalia Ferré^49^, Dariusz Gruszfeld^50^, Kathrin Gürlich^48^, Jean-Paul Langhendries^51^, Veronica Luque^49^, Enrica Riva^52^, Phillipp Schwarzfischer^48^, Martina Totzauer^48^, Elvira Verduci^52^, Annick Xhonneux^51^, Marta Zaragoza-Jordana^49^, Maarten Lindeboom^53^, Ameli Schwalber^54^, Nina Donner^54^, Rae-Chi Huang^55^, Rachel E. Foong^55,56^, Graham L. Hall^55,56^, Ashleigh Lin^55^, Jennie Carson^55^, Phillip Melton^57,58^, Sebastian Rauschert^55^

^1^Department of Pediatrics, Erasmus MC, University Medical Center Rotterdam, Rotterdam, the Netherlands. ^2^The Generation R Study Group, Erasmus MC, University Medical Center Rotterdam, Rotterdam, the Netherlands. ^3^Department of Child and Adolescent Psychiatry and Psychology, Erasmus MC, University Medical Center Rotterdam, Rotterdam, the Netherlands. ^4^Department of Psychology, Education and Child Studies, Erasmus School of Social and Behavioural Sciences, Rotterdam, the Netherlands. ^5^CIBER Epidemiología y Salud Pública (CIBERESP), Spain. ^6^ISGlobal, Barcelona, Spain. ^7^Universitat Pompeu Fabra (UPF), Barcelona, Spain. ^8^IMIM (Hospital del Mar Medical Research Institute), Barcelona, Spain. ^9^Institut d’Investigació Sanitària Pere Virgili (IISPV), Hospital Universitari Sant Joan de Reus, Reus, Spain. ^10^Biodonostia, Health research institute, San Sebastian, Spain. ^11^University of Basque Country, Spain. ^12^Epidemiology and Environmental Health Joint Research Unit, FISABIO–Universitat Jaume I–Universitat de València, Valencia, Spain. ^13^Faculty of Nursing and Chiropody, Universitat de València, Valencia, Spain. ^14^Conselleria de Sanitat, Valencia, Spain. ^15^Cancer Epidemiology Unit, Department of Medical Sciences, University of Turin, Turin, Italy. ^16^Institute of Developmental Sciences, Faculty of Medicine, University of Southampton, Southampton, United Kingdom. ^17^NIHR Southampton Biomedical Research Centre, University of Southampton and University Hospital Southampton NHS Foundation Trust, Southampton, United Kingdom. ^18^MRC Lifecourse Epidemiology Unit, University of Southampton, Southampton General Hospital, Southampton, United Kingdom. ^19^MRC Integrative Epidemiology Unit at the University of Bristol, Bristol, United Kingdom. ^20^NIHR Bristol Biomedical Research Centre, Bristol, United Kingdom. ^21^Population Health Science, Bristol Medical School, University of Bristol, Bristol, United Kingdom. ^22^Section of Epidemiology, Department of Public Health, University of Copenhagen, Copenhagen, Denmark. ^23^Bradford Institute for Health Research, Bradford Teaching Hospitals NHS Foundation Trust, Bradford, United Kingdom. ^24^University of Manchester, Manchester, United Kingdom. ^25^University of Groningen, University Medical Center Groningen, Genomics Coordination Center, Groningen, the Netherlands. ^26^University of Groningen, University Medical Center Groningen, Department of Genetics, Groningen, the Netherlands. ^27^Department of Epidemiology, University of Groningen, University Medical Center Groningen, Groningen, the Netherlands. ^28^Department of Social Medicine, Faculty of Medicine, University of Crete, Heraklion, Crete, Greece. ^29^Department of Preventive Medicine, Keck School of Medicine, University of Southern California, Los Angeles, CA, USA. ^30^Centre for Fertility and Health, Norwegian Institute of Public Health, Oslo, Norway. ^31^Division of Health Data and Digitalization, Norwegian Institute of Public Health, Oslo, Norway. ^32^Department of Genetics and Bioinformatics, Division of Health Data and Digitalisation, Norwegian Institute of Public Health, Oslo, Norway. ^33^Norwegian Institute of Public Health, Oslo, Norway. ^34^Université de Paris, Centre for Research in Epidemiology and Statistics (CRESS), INSERM, INRAE, Paris, France. ^35^ELFE Joint Unit, French Institute for Demographic Studies (INED), French Institute for Medical Research and H ealth (INSERM), French Blood Agency, Aubervilliers, France. ^36^Institut National d’Etudes Démographiques (INED), Aubervilliers, France. ^37^Université Grenoble Alpes, Inserm, CNRS, Team of Environmental Epidemiology Applied to Reproduction and Respiratory Health, IAB, Grenoble, France. ^38^Sorbonne Université, INSERM, Institut Pierre Louis d’ Epidemiologie et de Santé Publique (IPLESP), Equipe de Recherche en Epidémiologie Sociale (ERES), Paris, France. ^39^Center For Life-course Health research, Faculty of Medicine, University of Oulu, Oulu, Finland. ^40^Department of Epidemiology and Biostatistics, MRC-PHE Centre for Environment and Health, School of Public Health, Imperial College London, London, United Kingdom. ^41^Department of Life Sciences, College of Health and Life Sciences, Brunel University London, London, United Kingdom. ^42^Unit of Primary Health Care, Oulu University Hospital, OYS, Oulu, Finland. ^43^Infrastructure for Population Studies, Faculty of Medicine, University of Oulu, Oulu, Finland. ^44^Department of General Practice and Primary Health Care, University of Helsinki and Helsinki University Hospital, Helsinki, Finland. ^45^Folkhälsan Research Center, Helsinki, Finland. ^46^Obstetrics & Gynecology, Yong Loo Lin School of Medicine, National University of Singapore and National University Health System, Singapore. ^47^Singapore Institute for Clinical Sciences (SICS), Agency for Science and Technology (A*STAR), Singapore. ^48^Department of Pediatrics, Dr.von Hauner Children’s Hospital, University Hospital, LMU, Munich, Germany. ^49^Universitat Rovira i Virgili, IISPV, Tarragona, Spain. ^50^Neonatal Department, Children’s Memorial Health Institute, Warsaw, Poland. ^51^CHC St Vincent, Liège-Rocourt, Belgium. ^52^University of Milan, Milan, Italy. ^53^Department of Economics, VU University Amsterdam, Amsterdam, the Netherlands. ^54^Concentris Research Management GmbH, Fürstenfeldbruck, Germany. ^55^Telethon Kids Institute, Perth, Western Australia, Australia. ^56^School of Physiotherapy and Exercise Science, Curtin University, Perth, Western Australia, Australia. ^57^Curtin/UWA Centre for Genetic Origins of Health and Disease, School of Biomedical Sciences, The University of Western Australia, Australia. ^58^School of Pharmacy and Biomedical Sciences, Curtin University, Perth, Western Australia, Australia
