# Supplementary material for: The LifeCycle Project-EU Child Cohort Network: a federated analysis infrastructure and harmonized data of more than 250,000 children and parents
Source: Eur J Epidemiol. 2020 Jul 23;35(7):709–24. doi: 10.1007/s10654-020-00662-z (PMC7387322; doi:10.1007/s10654-020-00662-z)
Supplement: Supplementary file 1 — Supplementary material 1 (DOCX 25 kb) [file 10654_2020_662_MOESM1_ESM.docx]

**Supplementary Table S1: Study specific acknowledgements**

**ALSPAC**

We are extremely grateful to all of the families who took part in ALSPAC, the midwives for their help in recruiting them, and the whole ALSPAC team, which includes interviewers, computer and laboratory technicians, clerical workers, research scientists, volunteers, managers, receptionists and nurses.

**BIB**

The authors acknowledge that Born in Bradford is only possible because of the enthusiasm and commitment of the children and parents in Born in Bradford. We are grateful to all participants, health professionals and researchers who have made Born in Bradford happen.

**CHOP**

The authors would particularly like to thank all the cohort participants for their generous collaboration. Furthermore, thanks all persons who designed and conducted the study, entered the data, and participated in the data analysis and who are represented by the European Childhood Obesity Trial Study Group participants: B Koletzko, V Grote, M Totzauer, K Gürlich, P Schwarzfischer, N Aumüller, V Luque, M Zaragoza-Jordana, N Ferré, J Escribano, R Closa-Monasterolo, A Xhonneux, Jean-Paul Langhendries, E Verduci, E Riva, D Gruszfeld.

**DNBC**

The authors would like to thank the participants, the first Principal Investigator of DNBC Prof. Jørn Olsen, the scientific managerial team, and DNBC secretariat for being, establishing, developing and consolidating the Danish National Birth Cohort.

**EDEN**

The authors thank the cohort participants and the EDEN mother-child study group, whose members are: I. Annesi-Maesano, J.Y. Bernard, J. Botton, M.A. Charles, P. Dargent-Molina, B. de Lauzon-Guillain, P. Ducimetière, M. de Agostini, B. Foliguet, A. Forhan, X. Fritel, A. Germa, V. Goua, R. Hankard, B. Heude, M. Kaminski, B. Larroque†, N. Lelong, J. Lepeule, G. Magnin, L. Marchand, C. Nabet, F Pierre, R. Slama, M.J. Saurel-Cubizolles, M. Schweitzer, O. Thiebaugeorges.

**ELFE**

The authors are grateful to 1) the former members of the Elfe unit without whom the project would never have started: Henri Léridon, initiator and former Principal Investigator of the project, Stéphanie Vandentorren, Claudine Pirus, and Ando Rakotonirina; 2) the expertise and assistance of members of the unit for support functions, 3) all the researchers who contribute to the projects as members of the Elfe thematic groups and especially their coordinators; 4) all the field research assistants and interviewers; 5) and above all, all the Elfe families who have placed their confidence in us and given up their time to the study

**GECKO Drenthe**

The authors are grateful to the families who took part in the GECKO Drenthe study, the midwives, gyneacologists, nurses and GPs for their help for recruitment and measurement of participants, and the whole team from the GECKO Drenthe study.

**Generation R**

The authors gratefully acknowledge the contribution of participants, research collaborators, general practitioners, hospitals, midwives, and pharmacies in Rotterdam.

**HBCS**

The authors would particularly like to thank all the cohort participants for their generous collaboration.

**INMA**

The authors would particularly like to thank all the participants for their generous collaboration. The authors are grateful to Mireia Garcia, Maria Victoria Estraña, Maria Victoria Iturriaga, Cristina Capo and Josep LLuch for their assistance in contacting the families and administering the questionnaires.

**MoBa**

The authors are grateful to all the participating families in Norway who take part in this on-going cohort study.

**NFBC**

The authors would particularly like to thank all the cohort participants for their generous collaboration.

**NINFEA**

The authors thank all families participating in the NINFEA cohort.

**RAINE Study**

The authors would like to acknowledge the Raine Study participants and their families. The authors would also like to acknowledge the Raine Study Team for study co-ordination and data collection, and the NH&MRC for their long term contribution to funding the study over the last 29 years.

**RHEA**

The authors would particularly like to thank all the cohort participants for their generous collaboration.

**SWS**

The authors are grateful to the women of Southampton who gave their time to take part in the Southampton Women’s Survey and to the research nurses and other staff who collected and processed the data.

**Supplementary Table S2: Cohort study specific funding**

**ALSPAC**

Core funding for the Avon Longitudinal Study of Parents and Children (ALSPAC) is provided by the UK Medical Research Council and Wellcome (217065/Z/19/Z) and the University of Bristol. A comprehensive list of grants funding is available on the ALSPAC website (http://www.bristol.ac.uk/alspac/external/documents/grant-acknowledgements.pdf). DAL and AK work in a unit that is supported by the University of Bristol and UK Medical Research Council (MC_UU_00011/6) and DAL holds a European Research Council Advanced Grant (ERC grant agreement no 669545) and is a NIHR Senior Investigator (NF-0616-10102). The funders had no role in the design of the study, the collection, analysis, or interpretation of the data; the writing of the manuscript, or the decision to submit the manuscript for publication. The views expressed in this paper are those of the authors and not necessarily those of any funder.

**BIB**

BiB receives core infrastructure funding from the Wellcome Trust (WT101597MA) and a joint grant

from the UK Medical Research Council (MRC) and Economic and Social Science Research Council (ESRC) (MR/N024397/1). This study has received support from the British Heart Foundation (CS/16/4/32482), US National Institutes of Health (R01 DK10324), European Research Council

under the European Union's Seventh Framework Programme (FP7/2007-2013) / ERC grant agreement no 669545, and National Institute for Health Research ARC Yorkshire and Humber (NIHR200166. PMW receives funding from the National Institute for Health Research Applied Research Collaboration for Greater Manchester. The views expressed are those of the author(s), and not necessarily those of the NHS, the NIHR or the Department of Health and Social Care.

**CHOP**

The CHOP study has been carried out with partial financial support from the Commission of the European Community, specific RTD Programme "Quality of Life and Management of Living Resources", within the Fifth Framework Program (research grants no. QLRT-2001-00389 and QLK1-CT-200230582), the Sixth Framework Program (contract no. 007036), and Seventh Framework Programme (EarlyNutrition; grant agreement no. 289346), the EU H2020 project LIFECYCLE under grant no. 733206 and the European Research Council Advanced Grant META-GROWTH (ERC-2012-AdG – no.322605) and with financial support from Polish Ministry of Science and Higher Education (2571/7.PR/2012/2). This manuscript does not necessarily reflect the views of the Commission and in no way anticipates the future policy in this area. No funding bodies had any role in the study design, data collection and analysis.

**DNBC**

The Danish National Birth Cohort was established with a significant grant from the Danish National Research Foundation. Additional support was obtained from the Danish Regional Committees, the Pharmacy Foundation, the Egmont Foundation, the March of Dimes Birth Defects Foundation, the Health Foundation and other minor grants. The DNBC Biobank has been supported by the Novo Nordisk Foundation and the Lundbeck Foundation. Follow-up of mothers and children have been supported by the Danish Medical Research Council (SSVF 0646, 271-08-0839/06-066023, O602-01042B, 0602-02738B), the Lundbeck Foundation (195/04, R100-A9193), The Innovation Fund Denmark 0603-00294B (09-067124), the Nordea Foundation (02-2013-2014), Aarhus Ideas (AU R9-A959-13-S804), University of Copenhagen Strategic Grant (IFSV 2012), and the Danish Council for Independent Research (DFF – 4183-00594 and DFF - 4183-00152). AP is funded by a Lundbeck Foundation grant (R264-2017-3099)

**EDEN**

The EDEN study was supported by Foundation for medical research (FRM), National Agency for

Research (ANR), National Institute for Research in Public health (IRESP: TGIR cohorte santé 2008

program), French Ministry of Health (DGS), French Ministry of Research, INSERM Bone and Joint

Diseases National Research (PRO-A) and Human Nutrition National Research Programs, Paris-Sud

University, Nestlé, French National Institute for Population Health Surveillance (InVS), French

National Institute for Health Education (INPES), the European Union FP7 programmes (FP7/2007-

2013, HELIX, ESCAPE, ENRIECO, Medall projects), Diabetes National Research Program (through

a collaboration with the French Association of Diabetic Patients (AFD)), French Agency for

Environmental Health Safety (now ANSES), Mutuelle Générale de l’Education Nationale a

complementary health insurance (MGEN), French national agency for food security, French speaking

association for the study of diabetes and metabolism (ALFEDIAM).

**ELFE**

The Elfe cohort received funding from the National Research Agency Investment for the Future program [ANR-11-EQPX-0038]; French National Institute for Research in Public Health (IRESP TGIR 2009-2001 program); Ministry of Higher Education and Research; Ministry of Environment; Ministry of Health; French Agency for Public Health; Ministry of Culture; and National Family Allowance Fund.

**GECKO Drenthe**

The GECKO Drenthe birth cohort was funded by an unrestricted grant of Hutchison Whampoa Ld,

Hong Kong and supported by the University of Groningen , Well Baby Clinic Foundation Icare,

Noordlease, Paediatric Association Of The Netherlands and Youth Health Care Drenthe.

**Generation R**

The general design of the Generation R Study is made possible by financial support from the Erasmus

MC, University Medical Center, Rotterdam, Erasmus University Rotterdam, Netherlands

Organization for Health Research and Development (ZonMw), Netherlands Organisation for

Scientific Research (NWO), Ministry of Health, Welfare and Sport and Ministry of Youth and

Families. This project received funding from the European Union's Horizon 2020 research and innovation programme (LIFECYCLE, grant agreement No 733206, 2016; EUCAN-Connect grant agreement No 824989; ATHLETE, grant agreement No 874583). VJ received funding from a Consolidator Grant from the European Research Council (ERC-2014-CoG-648916). LD received funding from the European Union's Horizon 2020 co-funded programme ERA-Net on Biomarkers for Nutrition and Health (ERA HDHL) (ALPHABET project (no 696295; 2017), ZonMw The Netherlands (no 529051014; 2017)). The study sponsors had no role in the study design, data analysis, interpretation of data, or writing of this report.

**HBCS**

HBCS has been supported by grants from British Heart Foundation, Finska Läkaresällskapet, the Finnish Special Governmental Subsidy for Health Sciences, Academy of Finland, Samfundet Folkhälsan, Liv och Hälsa, Juho Vainio Foundation, Yrjö Jahnsson Foundation, The Diabetes Research Foundation, Finnish Foundation for Cardiovascular Research, the Signe and Ane Gyllenberg Foundation, EU FP7 (DORIAN) project number 278603, and EU Horizon 2020 (DynaHealth) project number 633595.

**INMA**

This study was funded by grants from the Instituto de Salud Carlos III (Red INMA G03/176) and the

Generalitat de Catalunya-CIRIT (1999SGR 00241). INMA-Valencia was funded by Grants from UE (FP7-ENV-2011 cod 282957 and HEALTH.2010.2.4.5-1), Spain: ISCIII (G03/176; FIS-FEDER: PI09/02647, PI11/01007, PI11/02591, PI11/02038, PI13/1944, PI13/2032, PI14/00891, PI14/01687, and PI16/1288; Miguel Servet-FEDER CP11/00178, CP15/00025, and CPII16/00051), and Generalitat Valenciana: FISABIO (UGP 15-230, UGP-15-244,and UGP-15-249). INMA-Gipuzkoa was funded by grants from the Instituto de Salud Carlos III (FISFIS PI06/0867, FISPS09/0009) 0867,Red INMA G03/176) and the Departamento de Salud del Gobierno Vasco (2005111093 and 2009111069) and the Provincial Government of Guipúzcoa (DFG06/004 and FG08/001). INM-Menorca was funded by grants from the Instituto de Salud Carlos III (Red INMA G03/176). This study was supported by funding from the European Community’s Seventh Framework Programme (FP7/2007-206) under grant agreement no 308333—the HELIX project. JJ holds Miguel Servet-II contract (CPII19/00015) awarded by the Instituto de Salud Carlos III (Co-funded by European Social Fund "Investing in your future"). ML has received funding from the European Union’s Horizon 2020 research and innovation programme under the Marie Skłodowska-Curie grant agreement No 707404. The opinions expressed in this document reflect only the author’s view. The European Commission is not responsible for any use that may be made of the information it contains. MC holds a Miguel Servet fellowship (CP16/00128) funded by Instituto de Salud Carlos III and co-funded by European Social Fund “Investing in your future". CW received a Sara Borrell fellowship (CD18/00132) from the Instituto de Salud Carlos III. RG was supported by funding from the Instituto de Salud Carlos III (PI14/00891 and PI17/00663) and Alicia Koplowitz Foundation 2017. ML has held a Miguel Servet-II contract (MSII16/00051) awarded by the Instituto de Salud Carlos III (Co-funded by European Social Fund "Investing in your future"). SL This study was supported by grants from Instituto de Salud Carlos III (FIS-FEDER: 13/1944, 16/1288 and 19/1338; Miguel Servet-FEDER: CP15/0025).

**MoBa**

The Norwegian Mother, Father and Child Cohort Study is supported by the Norwegian Ministry of Health and Care Services and the Ministry of Education and Research.

**NINFEA**

The NINFEA cohort was initially funded by the Compagnia SanPaolo Foundation and the Piedmont Region.

**NFBC1966 and NFBC1986**

NFBC1966 and 1986 have received financial support for data generation and for research and supporting staff from the Academy of Finland (grants numbers: 104781, 120315, 129269, 1114194, 24300796, 285547 (EGEA)); University Hospital Oulu, Biocenter, University of Oulu, Finland (grant number: 75617); NIHM (grant number: MH063706, Smalley and Jarvelin for NFBC1986 data collection), Juselius Foundation; NFBC1966 genotyping by NHLBI (grant number: 5R01HL087679-02] through the STAMPEED program [grant number: 1RL1MH083268-01); NIH/NIMH (grant number: 5R01MH63706:02); the European Commission: EURO-BLCS, Framework 5 award QLG1-CT-2000-01643 (for NFBC1986 data collection), ENGAGE project and grant agreement HEALTH-F4-2007 (grant number: 201413); EU H2020-HCO-2004 iHEALTH Action (grant number: 643774), EU H2020-PHC-2014 DynaHealth Action (grant number: 633595); ALEC Action (grant number: 633212); ERDF European Regional Development Fund (grant number: 539/2010 A31592); the Medical Research Council (MRC), UK (grant numbers: G0500539, G0600705, G1002319, MR/M013138/1), EU H2020-SC1-2016-2017 LifeCycle Action (grant number: 733206). The programme is currently funded by EU H2020-SC1-2016-2017 LifeCycle Action (grant number: 733206), EU-H2020 EDCMET (grant number: 825762), EU-H2020 EUCAN Connect (grant number: 824989), EU H2020-MSCA-ITN-2016 CAPICE Action Marie Sklodowska-Curie Grant (grant number: 721567) and by the Joint Programming Initiative a Healthy Diet for a Healthy Life (JPI HDHL; PREcisE Project, no 655) that is in the UK jointly funded by the Medical Research Council (MRC) and the Biotechnology and Biological Sciences Research Council (BBSRC) (grant number: MR/S03658X/1).

**RAINE Study**

The Western Australian Pregnancy Cohort (Raine Study) has been funded by program and project

grants from the Australian National Health and Medical Research Council, the Commonwealth

Scientific and Industrial Research Organisation, Healthway and the Lions Eye Institute in Western

Australia. The University of Western Australia (UWA), Curtin University, the Raine Medical

Research Foundation, the Telethon Kids Institute, the Women’s and Infant’s Research Foundation

(KEMH), Murdoch University, The University of Notre Dame Australia and Edith Cowan University

provide funding for the Core Management of the Raine Study. REF is a recipient of a National Health and Medical Research Council Early Career Fellowship.

**RHEA**

The "Rhea" project was financially supported by European projects (EU FP6-2003-Food-3-

NewGeneris, EU FP6. STREP Hiwate, EU FP7 ENV.2007.1.2.2.2. Project No 211250 Escape, EU

FP7-2008-ENV-1.2.1.4 Envirogenomarkers, EU FP7-HEALTH-2009- single stage CHICOS, EU FP7

ENV.2008.1.2.1.6. Proposal No 226285 ENRIECO, EU- FP7- HEALTH-2012 Proposal No 308333

HELIX) and the Greek Ministry of Health (Program of Prevention of obesity and neurodevelopmental

disorders in preschool children, in Heraklion district, Crete, Greece: 2011-2014; “Rhea Plus”: Primary

Prevention Program of Environmental Risk Factors for Reproductive Health, and Child Health: 2012-

15).

**SWS**

The SWS is supported by grants from the Medical Research Council, National Institute for Health

Research Southampton Biomedical Research Centre, British Heart Foundation, University of Southampton and University Hospital Southampton National Health Service Foundation Trust, and the European Union’s Seventh Framework Programme (FP7/2007-2013), project EarlyNutrition (grant 289346). Study participants were drawn from a cohort study funded by the Medical Research Council and the Dunhill Medical Trust. HMI's salary is paid by the UK Medical Research Council. Mark Hanson is supported by the British Heart Foundation.
